# Linear headache: a recurrent unilateral head pain circumscribed in a line-shaped area

**DOI:** 10.1186/1129-2377-15-45

**Published:** 2014-06-26

**Authors:** Yu Wang, Miao-Miao Tian, Xian-Hong Wang, Xiao-Qun Zhu, Ying Liu, Ya-Nan Lu, Qing-Qing Pan

**Affiliations:** 1 Department of Neurology, Epilepsy and Headache group, The First Hospital of Anhui Medical University, Jixi Road 218, Hefei 230022, China

**Keywords:** Linear headache, Epicrania fugax, Migraine, Cranial neuralgia, Trigeminal autonomic cephalalgias

## Abstract

**Background:**

A headache circumscribed in a line-shaped area but not confined to the territory of one particular nerve had ever been described in Epicrania Fugax (EF) of which the head pain is moving and ultrashort. In a 25-month period from Feb 2012 to Mar 2014, we encountered 12 patients with a paroxysmal motionless head pain restricted in a linear trajectory. The head pain trajectory was similar to that of EF, but its all other features obviously different from those of EF. We named this distinctive but undescribed type of headache linear headache (LH).

**Methods:**

A detailed clinical feature of the headache was obtained in all cases to differentiate with EF, trigeminal autonomic cephalalgias (TACs) and cranial neuralgia. Similarities and differences in clinical features were compared between LH and migraine.

**Results:**

The twelve LH patients (mean age 43.9 ± 12.2) complained of a recurrent, moderate to severe, distending (n = 9), pressure-like (n = 3) or pulsating (n = 3) pain within a strictly unilateral line-shaped area. The painful line is distributed from occipital or occipitocervical region to the ipsilateral eye (n = 5), forehead (n = 6) or parietal region (n = 1). The pain line has a trajecory similar to that of EF but no characteristics of moving. The headache duration would be ranged from five minutes to three days, but usually from half day to one day in most cases (n = 8). Six patients had the accompaniment of nausea with or without vomiting, and two patients had the accompaniment of ipsilateral dizziness. The attacks could be either spontaneous (n = 10) or triggered by noise, depression and resting after physical activity (n = 1), or by stress and staying up late (n = 1). The frequency of attacks was variable. The patients had well response to flunarizine, sodium valproate and amitriptyline but not to carbamazepine or oxcarbazepine. LH is different from EF, trigeminal autonomic cephalalgias (TACs) and cranial neuralgia, but it had couple of features similar to that of migraine.

**Conclusions:**

The clinical picture of LH might be a subtype of migraine, or represent a novel syndrome.

## Background

Head pain of intracranial or extracranial, such as cranial neuralgia, nummular headache, migraine and cluster headache, may be confined within a restricted area. But a headache area shaped in a linear trajectory which is not confined to the territory of one particular nerve has never been reported before the first report of Epicrania fugax (EF) by Pareja et al. in 2008 [1]. EF is characterized by brief pain paroxysms starting in a particular area of the posterior scalp, and rapidly radiating forwards along a linear trajectory to reach the ipsilateral forehead, eye, or nose in a few seconds [1-4]. And the paroxysmal pain may also move in an opposite direction along this trajectory [5-7]. Here, we described a paroxysmal head pain restricted in a linear trajectory similar to that in EF. Nevertheless, its all other features including motion, duration, accompaniments, and preventive response to therapeutic drugs are obviously different from those of EF, but apparently similar to those of migraine. The patients presented with a recurrent motionless distending or pressure-like head pain circumscribed in a line-shaped area distributing from occipital or occipitocervical region to ipsilateral nose, forehead or parietal region. The features of the headache are so distinctive but consistent that we are probably facing a novel syndrome which we named linear headache (LH) or a variant of some established headaches.

## Methods

In a 25-month period (Feb 2012 to Mar 2014), we encountered twelve patients with stereotypical headaches. All the patients complained of a paroxysmal headache in an area of linear trajectory from occipital or occipitocervical region to the ipsilateral nose, forehead or parietal region. A detailed history was obtained in all cases and a special attention was paid to medical history of head trauma or macroscopic changes of the scalp. Patients were asked to point out the exact location of the pain-affecting area and to delineate it in size and shape. A complete history of duration, intensity, and quality of the headache at baseline and during exacerbations, presence of nausea, vomiting, phonophobia and photophobia was recorded. A complete physical and neurological examination was performed in all cases. Inspection, palpation and sensory examination of the affecting area, as well as palpation of the supraorbital, infraorbital, minor occipital and greater occipital nerves were included in the examination. Routine blood work-up with erythrocyte sedimentation rate, computed tomography (CT) or magnetic resonance imaging (MRI) of the head and electroencephalogram (EEG) were performed in all cases to exclude any underlying disease. Diagnosis of other concomitant headache syndromes was based on the 2nd Edition of The International Headache Classification (ICHD-II) criteria [8].

## Results

Among the twelve reported patients (Table 1), six was male and six female. They were aged 23–65 years (mean 43.9 ± 12.2 years), and the age at onset of the headache paroxysms ranged from 21 to 63 years (mean 40.6 ± 12.0 years). Two patients (patient 2 and 11) had a history of migraine headaches. However, there was no temporal connection between the migraine headaches and the currently reported headaches in these two patients. Other ten patients had no other types of recurrent headaches. Apart from that, past medical history was unremarkable. Three patients were in one family in which patient 11 and 12 are the two sons of patient 6.

**Table 1:** Demographic and clinical features of patients with linear headache (LH)

	**Patient no.**					
	**1**	**2**	**3**	**4**	**5**	**6**
Sex,	Female	Female	Female	Male	Female	Female
Age, years	50	65	36	50	42	49
Age at onset (years)	47	63	36	50	39	37
Other headaches	None	Migraine	None	None	None	None
LH paroxysms						
Side of LH	Left	Left	Right	Right	Left	Right
Headache area	Occipital –forehead	Occipital -forehead	Occipitocervical -nose	Occipital -forehead	Occipitocervical -nose	Occipitocervical –nose
Duration^△^	Half day	Half day	10 min	Half day	3 hours-half day	2-3 days
Character	Pressure-like	Pressure-like	Distending	Distending	Distending	Unclear
Intensity*	6-7	6-7	8-9	7-8	6-7	7-8
Acompaniments	None	Nausea, vomiting	Nausea Chest lacerating	Ipsilateral dizziness	Ipsilateral diziness	Ipsilateral eye and nasion pain; nausea, vomiting
Frequency	1/day	1/day	2-3/day	1/day	1/4-5days increasing to 1-2/day	10-12/year
Triggering factors	−	−	−	−	−	Noise, depression, resting after physical activity
Interictal symptoms	None	None	Uncomfortable of occiput	None	None	None
Temporal pattern	With remission	Chronic	With remission	With remission	Chronic	Chronic
Length of symptomatic period	3 years	2 years	6 month	2 month	2 years	12 years
Therapy						
Medicine	Flunarizine	Flunarizine	Amitriptyline	Sodium valproate	Sodium valproate	Amitriptyline
Response	Prevent recurrence	Prevent recurrence	Frequency and intensity reduced	Frequency and intensity reduced	Prevent recurrence	Frequency and intensity reduced
	**Patient no.**					
	**7**	**8**	**9**	**10**	**11**	**12**
Sex,	Male	Male	Male	Female	Male	Male
Age, years	47	45	36	58	26	23
Age at onset (years)	45	41	34	52	22	21
Other headaches	None	None	None	None	Migraine	None
LH paroxysms						
Side of LH	Left	Left	Right	Right	Left	Left
Headache area	Occipital –forehead	Occipital -forehead	Occipital -forehead	Occipital –parietal	Occipitocervical -nose	Occipitocervical –nose
Duration^△^	5-6 min	5-6 min-12 hours	Half day-1 day	Half day	1-2 days	1-2 days
Character	Pulsating and distending	Pressure-like and distending	Distending, straining	Distending	Pulsating and distending	Pulsating and distending
Intensity*	7-9	6-8	7-9	6-7	7-9	7-8
Acompaniments	None	Nausea, vomiting	None	None	Nausea, vomiting	Nausea
Frequency	1/1-2 days increasing to 3-5/day	1/day	5-6 (2 weeks)/year^▲^	1-2 (1 week) /month^▲▲^	Occasional, 7/4 years	Occasional, 3/2 years
Triggering factors	Stress, staying up late	−	−	−	−	−
Interictal symptoms	Dizziness	Diziness	None	None	None	None
Temporal pattern	Chronic	Chronic	With remission	With remission	With remission	With remission
Length of symptomatic period	2 years	4 years	2 years	6 years	4 years	2 years
Therapy						
Medicine	Sodium valproate and flunarizine	Flunarizine	Amitriptyline and Flunarizine	Sodium valproate and flunarizine	−	−
Response	Prevent recurrence	Frequency and intensity reduced	Pain relieved	Pain relieved	−	−

*Visual Analogical Scale (0 = no pain; 10 = the worst pain imaginable); — = not applicable.

^△^Half day, 2–10 hours; One day, 10–24 hours; More than one day, >24 hours.

^▲^The headache attacked every day for consecutive two weeks (one cluster of episodes) and there were five to six clusters one year.

^▲▲^The headache attacked every day for consecutive one week (one cluster of episodes) and there were one to two clusters one month.

The clinical features are shown in Table 1. Headache paroxysms were strictly unilateral and almost invariably recurred on the same side. Five patients had symptoms on the right and seven patients on the left, while the attacks shifted sides in only one patient. Most patients reported gradual onset of pain among a linear trajectory of 5 mm to 10 mm in width, which linked the occipital or occipitocervical region with the ipsilateral nose, forehead or parietal region. No radiation or moving of the pain along the linear trajectory was reported in all patients. The pain attack would last for 5 minutes to 3 days, but usually for half day to 1 day in most cases. The pain character was distending or pressure-like in most cases, but pulsating in three patients who also had distending feature sometimes. All patients denied that the pain was epicranial but complained it intracranial. During the attack, accompanied nausea with or without vomiting was reported in six patients and ipsilateral dizziness reported in two patients, while no accompanied symptom reported in four patients. The paroxysms were usually spontaneous in ten cases, but triggered by stress and staying up late in one case, by noise, depression and resting after physical activity in another one case. During the interictal period, nine patients had no symptoms at all, the other three had mild dizziness of the head or uncomfortable feeling of the occiput. The frequency of attacks was variable, ranging from seven attacks in four years to five attacks per day. The temporal pattern of the attacks was chronic in five patients who had been having attacks for two to ten years without a remission. The remaining seven patients had a remission period lasting for two weeks to one year after a recurrent period.

With regard to examination, no skin change was noted along the painful linear trajectory either during the ictal period or during the interictal period in all patients, but a hyperaesthesia to light touch noted along the painful linear trajectory in three patients (patient 1, 2 and 5). Regional mild pain was noted in two patients while palpating the supraorbital or greater occipital nerves, but no abnormal sensation noted in all other patients. Neurological examination, MRI, EEG and blood tests were normal in all patients.

Ten of the twelve patients accepted medical treatment and had good responses. Two patients (patient 1 and 2) treated with flunarizine (5 mg twice a day) alone had the headache recurrence prevented, but got the headache recurred days after stopping taking flunarizine. Two patients treated with amitriptyline (25 mg twice a day) alone (patient 3 and 6) and one patient treated with flunarizine (5 mg twice a day) alone (patient 8) had the frequency and intensity of the headache reduced. Two patients treated with sodium valproate (500 mg twice a day) alone (patient 5) or together with flunarizine (5 mg twice a day) (patient 7) had the headache recurrence prevented. Carbamazepine (200 mg three times a day) usage failed to relieve the linear headache in three patients (patient 3, 7 and 8) who had short duration of linear headache. Among these three patients, patient 7 and 8 then accepted treatment with flunarizine (5 mg twice a day) alone and had the headache severity and frequency reduced, further, patient 7 accepted treatment with flunarizine (5 mg twice a day) in combined with sodium valproate (500 mg twice a day) and had the headache recurrence prevented.

## Discussion

Here we report twelve patients presenting with a previously undescribed headache with consistent features both intra-individually and inter-individually. The main feature of this headache is a paroxysmal head pain restricted in a linear trajectory linking the occipital or occipitocervical region and the ipsilateral nose or forehead region. This line-shaped trajectory is similar to that described in EF. Apart from the similar pain area, a linear trajectory, all other features are obviously different from those of EF but apparently similar to those of migraine. The clinical features of this new condition apparently constitute a new headache entity, and we have named it LH.

As LH pain is circumscribed in an area of linear shape and this line-shaped trajectory is similar to that of EF pain [1-7], we need to differentiate it from EF. On the other hand, the area of the LH pain is correspondent to the scalp area supplied by the supraorbital nerve (SON) and the greater occipital nerve (GON), we also need to differentiate it from trigeminal (TN) and occipital neuralgia (ON) and some trigeminal autonomic cephalalgias (TACs) including short-lasting unilateral neuralgiform headache attacks with conjunctival injection and tearing (SUNCT) and short-lasting unilateral neuralgiform headache attacks with cranial autonomic symptoms (SUNA). But the much loner pain duration of the LH makes it obviously different from EF, TN, ON, and SUNCT/SUNA. Apart from the similar pain area, all other features of LH are obviously different from those of EF (Table 2). The much longer duration (days) of motionless distending, pressure-like, and pulsating pain in LH paient is dramatically different from the ultrashort duration (less than 10 seconds) of moving stabbing or electric pain in EF patient. And the accompaniments of dizziness, nausea, vomiting and phonophobia in LH make it obviously different from EF which usually has no accompaniment. Features characteristic of all TACs are pain in the trigeminal distribution (usually in the first trigeminal distribution, V1) and ipsilateral cranial autonomic features [9]. Some SUNCT patients may have a combination of nausea which was the main accompaniments of LH, but they all have a migrainous biology, i.e. personal or family history of migraine [10]. Theoretically, it is possible that the V1 TN occurs simultaneously with the attack of ON in an occasional condition and thus the pain distribution is overlapped with the LH pain. TN or ON is usually characterized by paroxysms of brief but severe pain followed by asymptomatic periods without pain. But, in some patients, a constant dull background pain may persist and this dull pain stays within the usual anatomic boundaries of the trigeminal nerve distribution [11]. It is also possible that this dull pain was inaccurately described as pressure-like or distending by patients. However, the accompaniments of dizziness, nausea, and vomiting common in LH patients have never been observed in TN or ON patients. Hereby, we may conclude that LH is different from EF, SUNCT/SUNA and neuralgia, and this is further supported by its well response to flunarizine and sodium valproate which had never been shown effective in EF, SUNCT/SUNA and neuralgia.

**Table 2:** Differences between Linear Headache (LH) and Epicrania Fugax (EF) in demographic and clinical features

	**Epicrania fugax**[1]**,**[2]	**Linear headache**
Mean age at onset (years)	46	40
Age range at onset (years)	23-84	21 to 63
Female/Male ratio	Female > Male	Female = Male
Spread of pain	100%	No
Zig-zag trajectory	Occasional	No
Pain character	Electric, stabbing	Distending, pressure-like
Accompaniments	Occasional, ocular, nasal	Common, vestibular and gastrointestinal
Usual attack duration	1-2 s	Half day to one day
Range of duration	1-10s	5 minutes to 2 weeks
Severity of pain	Moderate	Moderate to severe
Triggers	Stess, staying up late, noise, depression, resting after physical activity	Touch on the specific area (stemming area)
Interictal pain or tenderness of the trajectory	Half patients	No
Interictal dizziness of the ipsilateral hemisphere	No	Common
Treatment requirement	Some cases	Most cases
Response to flunarizine	No report	Common
Response to sodium valproate	No report	Common
Response to carbamazepine and oxcarbazepine	Well response	No response

Recently, Serna-Candel et al. described three cluster headache (CH) patients whose typical CH attacks started with mild or moderate pain of dull or tightening character at the occipital region and gradually moved forward over 10 to 30 minutes, finally reached the ipsilateral orbital area, and the authors named this headache “ascending CH” [12]. The LH pain trajectory is the same as that of the antecedent pain in the “ascending CH”, and the pain quality also similar to that of “ascending CH”, though the LH pain was motionless. The long-lasting (half day to days) motionless moderate to severe pain may overwhelm the short-lasting mild moving pain at the beginning of LH. Recently, we reported two patients whose typical CH and migraine headache attacks started with an antecedent EF of which the pain trajectory is the same as those of LH pain and those of the antecedent symptom in ascending CH [5]. Thus, it might be considered that LH is also associated with CH or migraine headache. There are plenty of similarities between LH and migraine in clinical features including pain character, severity, accompaniments, triggering factors, average episode duration, treatment requirement and treatment response to different drugs as summarized in Table 3. Of interest is that the LH in two patients (patient 9 and 10) attacked in a style of “clustering episodes”. The headache attacked every day for consecutive two weeks (one cluster of episodes) and there were five to six clusters one year in patient 9, and the headache attacked every day for consecutive one week (one cluster of episodes) and there were one to two clusters one month in patient 10. It should be noted that these two patients had no accompaniment of migrainous features, such as nausea, vomiting, and phonophobia or photophobia, and it has ever been reported that the initial head pain of “ascending CH” had the pain trajectory and pain character same as that of LH [12], thus, the “clustering episodes” style of headache attacks might also be suggestive of CH. But the longer episodic duration and the lack of accompanied trigeminal autonomic symptom are more suggestive of migraine in these two patients, as the “clustering episodes” style of headache attacks in the LH patients is also commonly found in migraine patients [13-15]. We recently reported a patient who had recurrent ophthalmoplegia which started with a headache same as the LH, i.e. head pain circumscribed in a line-shaped area, and the headache had couple of features similar to that of migraine, such as past history of recurrent migraine attacks, accompaniments of nausea, vomiting, and phonophobia, response to flunarizine and sodium valproate. The presentations of this case are suggestive of a subtype of ophthalmoplegic migraine (OM) but not a recurrent painful ophthalmoplegic neuropathy (RPON) [16] though the term of OM had been replaced by RPON in the 3rd edition of the International Headache Classification (ICHD-3) [17]. These may evidence that LH is closely related to migraine or probably exists as a subtype of migraine. On the other hand, differences also exist between the LH and migraine. The mean age at onset of LH (43.9 ± 12.2 year old) is obviously older than that of migraine (22 ± 15) and the female to male ratio in LH (1:1) obviously different from those in migraine (2.16:1) (Table 3). In fact, the characters of the headache fullfilling the diagnostic criteria for migraine without aura was only found in six patients (patients 2, 4, 6, 9, 11 and 12) if we treated the unilateral line-shaped head pain as unilateral headache, one of the character for migraine diagnosis [17]. Three other patients’ headaches (patient 1, 5 and 10) had no migrainous features of pulsating, nausea or vomitting, and did not fullfill the diagnostic criteria for migraine without aura. And the headache duration of 5 to 10 minutes in three LH patients (patient 3, 7 and 8) seemly also make it hard to link the LH with migraine whose headache duration medians were reported as minimum of 12 hours, maximum of 48 hours with an average of 24 hours [18]. Thus, the LH may represent a novel syndrome but not a subtype of migraine. It might also be possible that the short-lasting line-shaped headache in the three patients is essentially different from the long-lasting LH pain in other patients whose presentations are more suggestive of a subtype of migraine. Of interest is that the LH patients without accompaniment of migrainous features also had well response to flunarizine treatment alone (patient 1), sodium valproate alone (patient 5) or to the combination of flunarizine and sodium valproate (patient 10). Even among the three other LH patients with short pain duration (patient 3, 7 and 8), one had the recurrent head pain prevented after using flunarizine and sodium valproate (patient 7) and another one had the pain frequency and intensity reduced after using flunarizine alone (patient 8). This feature of probably nice response to flunarizine seemly suggests a link between migraine and the LH without migrainous characteristics.

**Table 3:** Comparison between Linear Headache (LH) and Migraine in demographic and clinical features

	**Migraine **[19]^**Δ**^	**Linear headache**
Mean age at onset (years)	22 ± 15	40.6 ± 12.0
Age range at onset (years)	6-65	21-63
Female/Male ratio	141/65 = 2.16:1	6/6 = 1:1
Severity of pain	Moderate to severe [18]	Moderate to severe
Pain character		
Pressure-like	23.8%	3:12 = 25%
Distending/stretching	9.7%	7:12 = 58.3%
Throbbing/pulsating	31.6%	3:12 = 25%
Accompaniments		
Nausea	57.8%	6:12 = 50%
Vomiting	32%	4:12 = 33.3%
Diziness	17%	2:12 = 16.7%
Photophobia	51.9%	0%
Phonophobia	58.7%	0%
Average episode duration (hours)		
Less than 2 hours	23.4%	3:12 = 25%
Half day (2–10 hours)	41.4%	5:12 = 41.7%
One day (10–24 hours)	14.1%	4:12 = 33.3%
More than one day (>24 hours)	21.1%	4:12 = 33.3%
Triggered by		
Noise, depression and resting after physical activity	Yes	Yes
Stress, staying up late	Yes	Yes
Treatment requirement	Approximate half patients[20]	Most cases
Treatment		
Response to flunarizine	Most patients [21]	Common
Response to sodium valproate	Most patients [22], and recommendation with level A evidence [23]	Common
Response to carbamazepine	Possibably effective in some patients with no new evidence [23]	No response
Response to oxcarbazepine	No response [24]	No response

^Δ^apart from the references indicated in migraine column, all other data in this column are from reference [19].

The pathogenesis of LH is unknown. It is common for headache patients including migraineurs to localise their pain to a particular area of occipital, parietal, frontal, and orbital region [19]. Recently, there is key experimental data suggesting a probable role of ventroposteromedial (VPM) nucleus of the thalamus in localising a pain to a particular region [25]. However, it is unimaginable that the localization of a line-shaped pain area is integrated just in the thalamus without inputs from a peripheral line-shaped area of nociceptors though the migraine onset does not require the peripheral sensory inputs to activate the trigeminovascular system [26]. The cortical spreading depression (CSD)-induced long-lasting activation of meningeal nociceptors innervated by the fibers of SON or GON [27] has been accepted to be one of the original migraine pathophysiological processes leading to the activation of central trigemino-vascular neurons in the spinal trigeminal nucleus (C1-2) underlying migraine headache [28]. The onset of LH may also develop through this pathway as its clinical features are similar to those of migraine. Plenty of evidences have shown that CSD is a common therapeutic target for currently prescribed migraine prophylactic drugs [29]. Preventive effects for LH prophylaxis were observed after using drugs which had been shown able to suppress CSD [30,31], yet, no effect observed for the antiepileptic drugs carbamazepine and oxcarbazepine which had been shown effective in many types of neuropathic pain. Though the non-efficiency of carbamazepine and oxcarbazepine on LH was observed only in two patients, this is consistent with their lack of efficacy on migraine [24,29] as well as on CSD [32,33]. On the other hand, recent research has provided experimental data implicating that complex meningeal immuno-vascular interactions, in particular, the interplay between proinflammatory cytokines, the meningeal vasculature and immune cells can lead to an enhancement of meningeal nociceptor responses [34]. Hereby, we propose that meningeal nociceptors in a line-shaped area parallel to the superior sagital sinus (SSS) is sensitized due to meningeal immuno-vascular interactions and thus prone to be activated by CSD in LH patients, resulting in a line-shaped cephalalgia through migraine pain pathway. In some conditions, CSD may activate wider area of meningeal nociceptors leading to migraine attacks in LH patients. This is seemly supported by the coexistence of migraine headaches in some patients with LH.

## Conclusions

LH presents an undescribed, clear-cut clinical picture that may be distinguished from other headaches. It is basically characterized by recurrent unilateral pain circumscribed in a line-shaped area from the occipital or occipitocervical region to the ipsilateral nose or forehead region, accompanied with or without nausea, vomitting and dizziness. LH might be a subtype of migraine, but might also represent a novel syndrome. At this stage, no definite explanation can be provided, but the clinical features point to a pathophysiological pathway similar to that of migraine. Further observations are required for a definitive characterization of these headaches, and the study of the LH pathophysiology may help to elucidate the mechanisms of migraine.

## Abbreviations

EF: Epicranial fugax; LH: Linear headache; CHD: International classification of headache disorders; MRI: Magnetic resonance imaging; CT: Computed tomography; EEG: Electroencephalogram; SON: Supraorbital nerve; GON: Greater occipital nerve; TN: Trigeminal neuralgia; ON: Occipital neuralgia; TACs: Trigeminal autonomic cephalalgias; SUNCT: Short-lasting unilateral neuralgiform headache attacks with conjunctival injection and tearing; SUNA: Short-lasting unilateral neuralgiform headache attacks with cranial autonomic symptoms; CH: Cluster headache; RPON: Recurrent painful ophthalmoplegic neuropathy; OM: Ophthalmoplegic migraine; CSD: Cortical spreading depression; SSS: Superior sagital sinus.

## Competing interests

The authors declare that they have no competing interests.

## Authors’ contributions

YW interviewed, diagnosed and treated the patients, interpreted the data and drafted the manuscript for content. All other authors listed contributed to the follow-up of the patients and literature reviewing. All authors read and approved the final manuscript.
